# Specialties and Specialists

**Published:** 1880-04

**Authors:** 


### EDITORIAL DEPARTMENT.

"If thou hast truth to utter, speak;
And leave the rest to God."

Specialties and Specialists.—Should the prevailing tendency among certain physicians to adopt and announce themselves as practising particular branches of the profession, cause them to withdraw from general practice entirely, the result would likely be to have here, as in England, a larger number of distinguished men in the profession. It is obvious that a physician who cultivates a special branch or department of the science of medicine, for which he might be better suited by nature and inclination, would be far more certain to succeed, and attain even to eminence, in it than he would likely do were he to remain in general practice. In addition to this, the field of his labors being reduced, and greater opportunity and leisure afforded for its investigation and culture than is ordinarily enjoyed by the general practitioner, he would consequently, or at least ought to, become an expert in his single branch. To this end such departures should be encouraged rather than hindered by the neighboring brethren of the profession. But the choice of a specialty having once been made it should in honor be rigidly adhered to alone in future practice, as in this way only can the specialist hope for, or indeed have, any right to expect assistance and encouragement from the general practitioner; and just here it is pleasant to record the fact that there are honorable men in the specialty ranks who do not, nor cannot so far forget their duty to their brethren engaged in general practice as to trespass again upon the domain of their labors. But unfortunately these constitute a small portion, probably not even a moiety, of those styling themselves specialists. There are some in fact who fall immeasurably below the standard that governs the upright and honorable specialist, and who make the endorsement and recommendation of the general practitioner, of them as specialists, useful in procuring patients with every form of disease for their offices, and also visit them at their homes or elsewhere. Nature is not so lavish in the bestowal of her gifts as to fit this latter class for a special branch of practice in a high degree, and enable them to compete with their brethren in general professional work at the same time. Cleverness is frequently found, and even a fair share of talent is not wanting in the ranks of the specialists, but genius is comparatively a rare quality in any calling. Let the specialist therefore adhere strictly to his department, and he will find his perhaps less richly gifted brother, in general practice, throwing more cases into his hands than he would be likely to obtain by "running with the hare and following with the hounds" at one and the same time. In other words, let honesty and fair dealing characterize his expressions and conduct toward those engaged in general practice, or else take in his sign as a specialist, and defraud his brother of his patients no longer under false pretenses, by arrogating superior qualities or qualifications to himself.

